# The Modified Selenenyl Amide, M-hydroxy Ebselen, Attenuates Diabetic Nephropathy and Diabetes-Associated Atherosclerosis in ApoE/GPx1 Double Knockout Mice

**DOI:** 10.1371/journal.pone.0069193

**Published:** 2013-07-16

**Authors:** Sih Min Tan, Arpeeta Sharma, Derek Y. C. Yuen, Nada Stefanovic, Guy Krippner, Govindasamy Mugesh, Zhonglin Chai, Judy B. de Haan

**Affiliations:** 1 Oxidative Stress Group, Diabetic Complications Division, Baker IDI Heart and Diabetes Institute, Melbourne, Australia; 2 Medicinal Chemistry, Baker IDI Heart and Diabetes Institute, Melbourne, Australia; 3 Department of Inorganic & Physical Chemistry, Indian Institute of Science, Bangalore, India; 4 Proliferation and Fibrosis Group, Diabetic Complications Division, Baker IDI Heart and Diabetes Institute, Melbourne, Australia; University of Louisville, United States of America

## Abstract

Seleno-organic glutathione peroxidase (GPx) mimetics, including ebselen (Eb), have been tested in *in vitro* studies for their ability to scavenge reactive oxygen and nitrogen species, including hydrogen peroxide and peroxynitrite. In this study, we investigated the efficacies of two Eb analogues, m-hydroxy ebselen (ME) and ethanol-ebselen (EtE) and compared these with Eb in cell based assays. We found that ME is superior in attenuating the activation of hydrogen peroxide-induced pro-inflammatory mediators, ERK and P38 in human aortic endothelial cells. Consequently, we investigated the effects of ME in an *in vivo* model of diabetes, the ApoE/GPx1 double knockout (dKO) mouse. We found that ME attenuates plaque formation in the aorta and lesion deposition within the aortic sinus of diabetic dKO mice. Oxidative stress as assessed by 8-OHdG in urine and nitrotyrosine immunostaining in the aortic sinus and kidney tubules, was reduced by ME in diabetic dKO mice. ME also attenuated diabetes-associated renal injury which included tubulointerstitial fibrosis and glomerulosclerosis. Furthermore, the bioactivity of the pro-fibrotic cytokine transforming growth factor-β (TGF-β) as assessed by phospho-Smad2/3 immunostaining was attenuated after treatment with ME. TGF-β-stimulated increases in collagen I and IV gene expression and protein levels were attenuated by ME in rat kidney tubular cells. However, in contrast to the superior activity of ME in *in vitro* and cell based assays, ME did not further augment the attenuation of diabetes-associated atherosclerosis and renal injury in our *in vivo* model when compared with Eb. In conclusion, this study strengthens the notion that bolstering GPx-like activity using synthetic mimetics may be a useful therapeutic strategy in lessening the burden of diabetic complications. However, these studies highlight the importance of *in vivo* analyses to test the efficacies of novel Eb analogues, as *in vitro* and cell based assays are only partly predictive of the *in vivo* situation.

## Introduction

Oxidative stress is recognised as playing a major role in the pathophysiology of disease, with evidence for its contribution to processes such as inflammation, fibrosis, and atherosclerotic lesion formation [1]. The two-electron reactive oxygen and nitrogen species (RO/NS) such as hydrogen peroxide and peroxynitrite (PN, ONOO^−^) are now considered more damaging than one-electron radicals, with greater disease-mediating capabilities [2]. Peroxynitrite is formed in biological reactions through the diffusion-controlled interaction of superoxide radicals (O_2_ **^.^** ^−^) with nitric oxide (NO**^.^**) and is involved in DNA [3] and lipid damage [4] as well as the nitrosative damage of proteins via the formation of nitrotyrosine adducts [5], [6]. Hydrogen peroxide mediates adverse cellular consequences at supraphysiological concentrations by enhancing processes such as apoptosis and necrosis [7], [8], and affecting important pro-atherogenic pathways such as the JNK and MAP-kinase pathways [9], [10], [11]. Given the injurious nature of these two-electron reactive species, attention has focused on antioxidants to lower the concentration of these RO/NS to avert disease. Recent interest has turned to the benefits of bolstering endogenous antioxidant-like defences, in particular that of the naturally occurring glutathione peroxidase (GPx) enzymes, since these antioxidants efficiently remove two-electron reactive species such as hydrogen and lipid peroxides as well as peroxynitrites [12]. The development of small synthetic compounds that mimic the activities of the GPx enzymes have received considerable attention for their potential to lessen prevailing RO/NS and thereby reverse or avert disease [13], [14].

Diabetes mellitus is one such disease where RO/NS cause cellular injury, in particular oxidative and nitrosative damage associated with its complications of atherosclerosis and kidney injury [15], [16]. Renal and vascular dysfunction share several common underlying pathogenic mechanisms, with oxidative stress and systemic inflammation contributing to both diabetic pathologies [17], [18], [19]. Indeed, the effective targeting of oxidative stress in diabetic patients could lead to a reduction in atherosclerosis and kidney damage. Furthermore, diabetic patients often display atherosclerosis and kidney failure as co-morbidities, highlighting the potential of a targeted antioxidant mono-therapy effective against both conditions.

**Figure 1:**
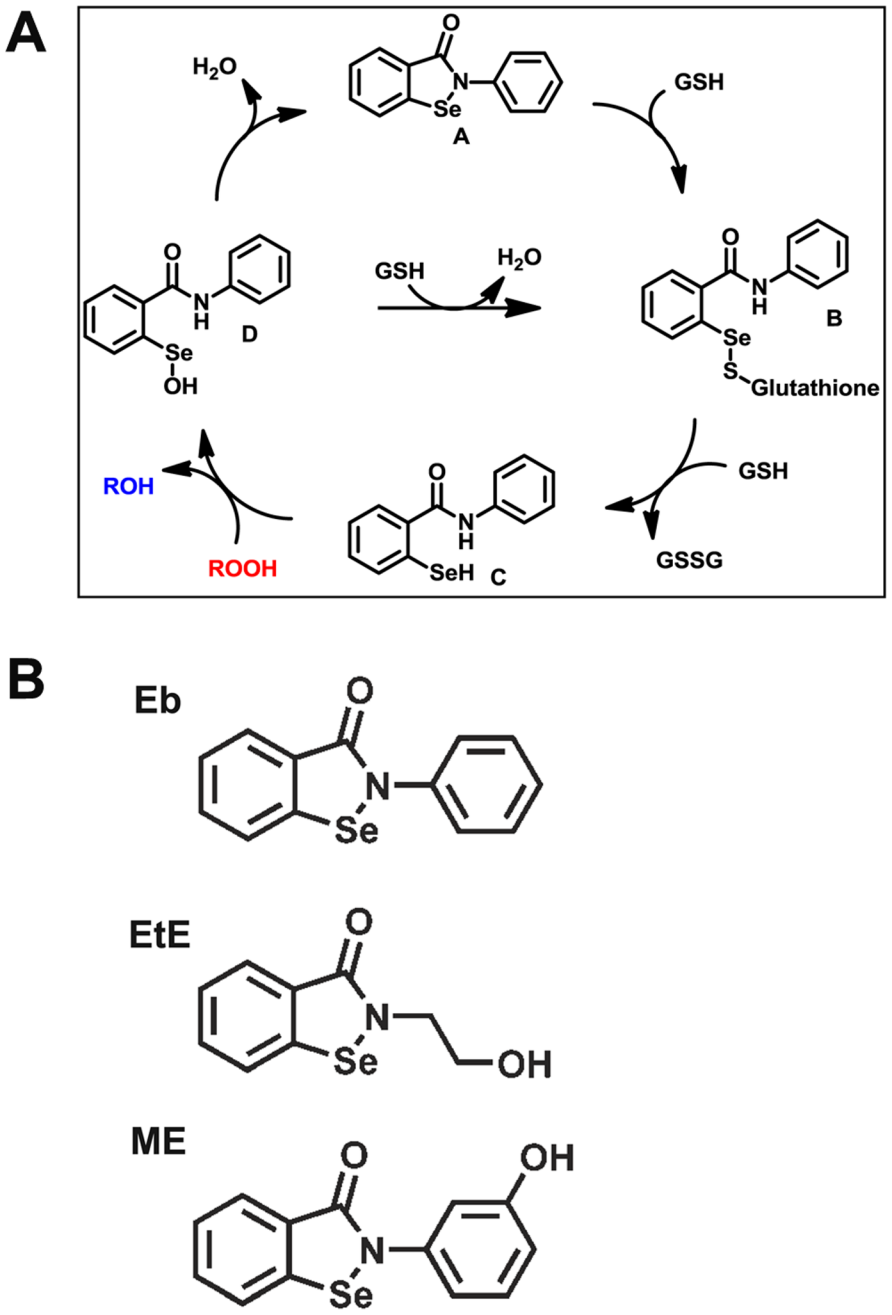
Proposed catalytic mechanism of ebselen and the structure of ebselen and novel analogues. **A)** GPx cycle of ebselen; In a first step, selenium within the active site of ebselen (A) interacts with one glutathione molecule to form the selenenyl sulphide, ebselen-S-GSH (B). Interaction with another GSH produces the active ebselen-selenol (C), which then interacts with biological peroxides to produce ebselen-selenenic acid (D). This moiety in turn can either interact with GSH to regenerate ebselen-S-GSH (B) or via the formation of water, the selenenyl amide, ebselen (A) is regenerated. GSH, reduced glutathione; GSSG, oxidised glutathione dimer; ROOH represents biological peroxides. **B)** Structures of ebselen (Eb), ethanol-ebselen (EtE) and m-hydroxy ebselen (ME).

Recently, we have shown in *in vitro* studies that the lipid-soluble low molecular weight seleno-organic GPx mimetic, ebselen (Eb), as well as a range of Eb analogues, act as efficient catalysts in the decomposition of peroxynitrite [20]. Our studies were in agreement with previous investigations showing the interaction of Eb with PN to be 3–4 orders greater than that observed with other antioxidants such as cysteine, ascorbate or methione [21]. Importantly, our study suggested that the efficacy of Eb could be further enhanced by suitable substitutions at its phenyl ring. Indeed we showed that the addition of a hydroxyl group in the meta- or 3- position of the phenyl ring of Eb (m-hydroxy ebselen or ME) reduced the IC_50_ for the inhibition of PN-mediated nitration of L-tyrosine by approximately 2.5 fold compared with Eb, suggesting that the modified analogue ME behaves in a superior fashion to Eb in our *in vitro* assays [20]. Indeed, we have previously shown that any substituent that enhances the nucleophilic attack of a thiol at the sulfur centre in the selenenyl sulfide state (B, Figure 1A), enhances the antioxidant potency of the compound by reducing the barrier for the formation of the active species selenol (C, Figure 1A) [22].

However, it becomes important to test these compounds in cellular models since selenium compounds showing good catalytic activity in *in vitro* experiments cannot be assumed to show good antioxidant behavior *in vivo*. For this reason, we have now compared the efficacy of two modified Eb analogues [ME and ethanol-ebselen (EtE), an analogue with an ethanol substitution on the nitrogen atom of the 5-membered ring of Eb; see Figure 1B] with Eb in cell based assays. Based on our previous *in vitro* studies [20], [23] and our current cell culture studies, we chose one analogue for further testing in animal models of diabetes. Consequently, we have now compared the efficacy of ME with Eb in the reduction of oxidative stress, atherogenic lesions and the ability to lessen endpoints of kidney disease in our well established diabetic model, the diabetic ApoE/GPx1 double knockout (dKO) mouse. Our rationale for these *in vivo* experiments was two-fold; firstly to establish whether ME lessened oxidative stress and limited the development of diabetic complications in the diabetic ApoE/GPx1 dKO mouse [24], and secondly to confirm our *in vitro* data that this modification of Eb rendered the behavior of ME superior to that of Eb [25] *in vivo*. Our results have shown that the modified Eb analogue, ME, attenuates oxidative stress, atherosclerosis and the hallmarks of diabetic nephropathy in the diabetic ApoE/GPx1 dKO mouse. However, our results highlight that the superior *in vitro* activity of this modified Eb analogue does not guarantee its superior *in vivo* function when compared with the parent compound Eb.

## Materials and Methods

### Ebselen and its Analogues

The Eb analogues, ME and EtE, were synthesized as detailed previously (compounds 18 and 20 respectively, as detailed in Bhabak et al. [23]). Ebselen [2-phenyl-1,2-benzisoselenazol-3(2H)-one] was purchased from Sapphire Bioscience, NSW, Australia. For *in vitro* NRK cell-based assays, both Eb and its analogues were made up as a 100 mM stock in DMSO. On the day of treatment, the compounds were further diluted with DMSO to produce a 1.25 mM working solution. To 500 µl of medium in a 6-well culture plate, 1 µl of 1.25 mM Eb or analogue was added, giving a final drug concentration of 2.5 µM. Control wells were treated in the same manner except that 1 µl of DMSO was added instead of drug treatment. For *in vitro* HAEC cell-based assays, both Eb and its analogues were made up as a 20 mM stock in DMSO. On the day of treatment, the compounds were further diluted with DMSO to produce a 20 µM working solution. To 2 ml of medium in a 6-well culture plate, 1 µl of 20 µM Eb or analogue was added, giving a final drug concentration of 0.01 µM. Control wells were treated in the same manner except that 1 µl of DMSO was added instead of drug treatment.

### In vitro Assays in HAEC and NRK Cells

#### Human Aortic Endothelial cells (HAEC)

Cells at passage 4–13 (Cell Application, San Diego, CA, USA) were maintained in endothelial cell media (EGM-2 bullet kit, Lonza, MD, USA) and seeded into 6-well culture plates, pre-coated with 0.2% gelatin. When cultures were 80% confluent, cells were serum deprived for 18 h before experiments were conducted. Cells were pre-treated with 0.01 µM Eb or the analogues for 30 min after which hydrogen peroxide (H_2_O_2_; 200 µM, Merck) was added for an additional 30 min in the presence of Eb or the analogues. In separate experiments, control cells were either not treated or treated with 200 µM H_2_O_2_ for 30 min; DMSO (vehicle control) or 0.01 µM drug alone for 60 min. In previously published studies, we had determined that treatment with 0.01–0.03 µM Eb was optimal for the inhibition of JNK and IKK activation, whilst significantly inhibiting Nox2 and TNF-α, and without compromising cell viability [26].

#### Normal Rat Kidney (NRK) cells

Rat kidney tubular epithelial cell line (NRK52E) was obtained from the American Tissue Culture Collection (Rockville, MD, USA) and maintained in Dulbecco’s modified Eagle medium containing 10% serum and 25 mmol/l glucose. Cells were starved in 0.1% FBS medium and pre-treated with ME or Eb at 2.5 µM or vehicle (DMSO) for 4 h before being stimulated with TGF-β_1_ at 10 ng/ml (recombinant human, R&D Systems, Minneapolis, USA) for 72 h. In pilot studies, Eb was used at 0.5, 1.0, 2.5, 5.0, 10 and 20 µM. We determined that 2.5 µM Eb was optimal for the inhibition of TGF-β-stimulated collagens expression, without compromising cell viability (data not shown). Thus, in this study 2.5 µM of Eb and ME was used in all *in vitro* experiments using NRK cells. Thereafter the cells were collected for either RNA extractions using TRIzol^R^ Reagent (Invitrogen Life Technologies) and analysis by RT-qPCR analysis, or for protein lysates and analysis by Western blots as described previously [25].

### Animals

Animal studies were approved by the AMREP Animal Ethics Committee and investigations conformed to National Health and Medical Research Council (NHMRC; Australia) guidelines (Permit number: E/1034/2010/B). All efforts were made to minimize suffering and diabetic animals had their bedding changed daily to reduce wetness per box. Eight-week old male ApoE/GPx1 dKO mice were rendered diabetic by two intraperitoneal injections of streptozotocin (STZ, Sigma, USA) at 100 mg/kg/day for two consecutive days. Due to the more resistant nature of the β-islet cells of the ApoE/GPx1 mouse to STZ killing, we have previously optimised the dosage of STZ to produce a sustained blood glucose level of around 25 mM for the duration of the study [24], [25]. Mice injected with citrate buffer served as non-diabetic controls. At 10-week of age, diabetic and non-diabetic mice were randomised to receive treatment of Eb or ME at 10 mg/kg twice daily by gavage. Since we had previously shown an approximately 50% reduction in plaque in the diabetic ApoE/GPx1 dKO mouse model using this drug regimen [25], we specifically chose this dose for both Eb and ME so that we could compare the results obtained after ME treatment with the expected reductions after Eb treatment. We hypothesized that the more efficacious ME would further reduce the extent of lesions compared with the parent compound, Eb. After 12 weeks of treatment, urine was collected for kidney function assessment before the mice were killed by lethal injection of 2,2,2-tribromoethanol (Sigma, USA) and direct puncture of the right ventricle to obtain blood. Aortas and kidney tissue were then collected and fixed in 10% neutral buffered formalin (NBF), whilst the heart sinuses were frozen in OCT after fixation in periodate-lysine-paraformaldehyde (PLP), for subsequent histological analyses. Kidney cortex and medulla were separated and snap frozen in liquid nitrogen for subsequent RNA extraction.

### Evaluation of Atherosclerotic Lesion

The *en face* analysis of atherosclerotic lesions was conducted as described previously [24], [26], after staining with Sudan IV-Herxheimer’s solution (BDH, Poole, UK), and photographs of opened aortas were digitised using a dissecting microscope (Olympus SZX9; Olympus Optical, Tokyo, Japan) and a digital camera (Axiocam colour camera; Carl Zeiss, North Ryde, NSW, Australia). Plaque area was calculated as the proportion of aortic intimal surface area occupied by red stained plaque (Adobe Photoshop v 6.0.1; Adobe Systems, Chatswood, NSW, Australia).

Lesions within the sinus region were visualised after staining of 10 µm sections with Oil Red O staining as described previously [24], [26]. The lesional area within the sinus region was quantitated by determining the total plaque area using Image Pro Plus software.

### Kidney Function Assessment

Kidney function was assessed by the measurement of urinary albumin to creatinine ratio (ACR). Urinary albumin excretion was measured by a mouse albumin ELISA kit according to manufacturer’s protocols (Bethyl Laboratories, USA). Urinary creatinine was measured by high-perfomance liquid chromatography (HPLC) method as detailed previously [25]. Albumin to creatinine ratio was then calculated by dividing concentration of albumin (mg/L) to concentration of creatinine (mmol/L) in the urine, and expressed as mg/mmol.

### Histological Assessment of Kidney Pathology

For all histological and immunohistochemical staining, six to 12 kidneys were analysed per treatment group (n = 6 to 12 animals per group). For each treatment group, 15–25 glomeruli (at 400× magnification) or at least 10 random non-overlapping fields (at 200× magnification) of the cortex were imaged for quantitation. All image analyses were performed in a blinded manner.

#### Tubulointerstitial fibrosis

Kidney sections (4 µm) were stained with Masson’s trichrome for the examination of tubulointerstitial matrix accumulation. Tissue sections were pre-fixed in pre-heated Bouin’s solution at 65°C for 30 min. Sections were then covered with Weigert’s iron haematoxylin for 10 min and washed. Slides were placed in 1% Brilliant Crocien (Amber Scientific, WA, Australia) for 15 min for cytoplasmic staining and rinsed in 1% phosphotungstic acid. Sections were then incubated in 0.5% Aniline Blue (ProSciTech, QLD, Australia) in 1% acetic acid to stain for collagen for a further 15 min. Finally, slides were rinsed with 1% acetic acid, dehydrated with 100% ethanol and xylene and coverslipped with DPX (DePeX; Merck, Vic, Australia). All sections were examined under light microscopy (Olympus BX-50; Olympus Optical) and digitised with a high-resolution camera. Proportional areas of matrix (blue staining) were quantitated with imaging software (Image-Pro Plus, v6.0).

#### Mesangial area

Kidney sections (3 µm) were stained with periodic-acid Schiff (PAS). Mesangial area was analysed from digital images of glomeruli using Image-Pro Plus v6.0 (Media Cybernetics, Bethesda, MD, USA) and expressed as percentage of PAS-stained area per glomerular cross sectional area (gcs).

#### Glomerulosclerosis

The degree of sclerosis (extent of mesangial matrix accumulation and cellularity) in each glomerulus was subjectively graded on a scale of 0–4 with grade 0 = normal; grade 1 = sclerotic area up to 25% (minimal); grade 2 = sclerotic area 25–50% (moderate); grade 3 = sclerotic area 50–70% (moderate to severe), and grade 4 = sclerotic area 75–100% (severe). The glomerulosclerosis index (GSI) was then calculated using the following formula: GSI = (1×n_1_)+(2×n_2_)+(3×n_3_)+(4×n_4_)/n_0_+ n_1_+ n_2_+ n_3_+ n_4_, where n_x_ is the number of glomeruli in each grade of glomerulosclerosis.

### Immunohistochemistry

Paraffin sections of kidney (4 µm) and frozen aortic sinus sections (10 µm) were used to stain for nitrotyrosine (rabbit polyclonal, Chemicon, MA, USA), which is a marker for peroxynitrite-induced damage in the tissue. The bioactivity of transforming growth factor-β (TGF-β) was assessed by measuring the activation of its downstream mediator, phosphorylated Smad2, using rabbit polyclonal antibody which detect the levels of Smad2/3 only when dually phosphorylated at Ser465 and Ser467 (Cell Signaling, MA, USA). Podocytes were identified using a rabbit monoclonal antibody against Wilms Tumor Protein (WT1; Abcam, MA, USA), which serves as a marker for mature podocyte nuclei [27]. The deposition of collagen IV surrounding the tubulointerstitium was assessed using a goat anti-type IV collagen antibody (Southern Biotech, Birmingham, AL, USA).

Immunohistochemical methods are briefly as follows. After hydration of sections, endogenous peroxidases were inactivated with 3% H_2_O_2_. Paraffin sections were incubated with protein blocking agent prior to application of the primary antibody. Primary antibody was left on overnight at 4°C. This was followed by incubation with biotinylated secondary antibody for 10 min at room temperature. Sections were then incubated with Vectastain ABC reagent (Vector Laboratories, CA, USA). Peroxidase activity was identified by reaction with 3,3′-diaminobenzidine tetrahydrochloride (Sigma-Aldrich Pty. Ltd, NSW, Australia). Sections were counterstained with hematoxylin. All sections were examined under light microscopy (Olympus BX-50; Olympus Optical) and digitised with a high-resolution camera. All digital quantitation (Image-Pro Plus, v6.0) and assessments were performed in a blinded manner.

### 8-Hydroxy-2-Deoxy Guanosine EIA

8-hydroxy-2-deoxy guanosine (8-OHdG) was measured in urine by a commercially available EIA kit (StressMarq Biosciences Inc, Victoria, BC, Canada). Urine was diluted 1∶100 for non-diabetic mice and 1∶10 for diabetic mice and the assay was performed as per the manufacturer’s instructions. Urinary 8-OHdG was normalised relative to urinary creatinine which is measured by HPLC as described above, and expressed as µg/mmol.

### Quantitative Reverse Transcription-Polymerase Chain Reaction (qRT-PCR)

RNA from mouse kidney cortex and NRK cells was extracted using TRIzol^R^ Reagent according to previously published methods [25]. Contaminating DNA was removed after treatment with DNA-free™ DNAse according to the manufacturer’s specifications (Ambion Inc, Austin, USA). DNA-free RNA was reverse transcribed into cDNA using the Superscript First Strand Synthesis System according to the manufacturer’s specifications (Life Technologies BRL, Grand Island, NY). Gene expression was analysed by real-time quantitative RT-PCR using the Taqman system based on real-time detection of accumulated fluorescence (ABI Prism 7700, Perkin-Elmer Inc., Foster City, CA, USA). Fluorescence for each cycle was analyzed quantitatively by an ABI Prism 7700 Sequence Detection System (Perkin-Elmer, PE Biosystems). Gene expression was normalised relative to the expression of the housekeeping gene 18S ribosomal RNA (18S rRNA Taqman Control Reagent kit; ABI Prism 7700) that was multiplexed together with the gene of interest. Amplifications were performed with the following time course: 50°C for 2 min, 95°C for 10 min, followed by 50 cycles of 94°C for 20s and 60°C for 1 min. The nucleotide sequences of rat *collagen I* primers and probes were as follows: 6-FAM (probe) CCTTCCTGCGCCTGA; forward primer TGCCGATGTCGCTATCCA; and reverse primer TCTTGCAGTGATAGGTGATGTTCTG. The probes and primers for rat *collagen IV* were: 6-FAM (probe) ATTTGCGTAACTAACACACC; forward primer CACTATGAAAACCGTAAAGTGCCTTA; and reverse primer GCAAACAGAGGCCAACGAA. For mouse *collagen IV* probes and primers: 6-FAM (probe) CAGTGCCCTAACGGT; forward primer GGCGGTACACAGTCAGACCAT; and reverse primer GGAATAGCCGATCCACAGTGA.

### Western Blot Analysis of NRK Cells

Forty-five micrograms of protein from each sample was separated by electrophoresis on 10% SDS polyacrylamide gels at 150V. The proteins were then transferred onto nitrocellulose membranes using wet transfer and incubated in blocking buffer [5% bovine serum albumin in Tris-buffered saline with 0.05% Tween (TBST)] for 2 hr. Membranes were then subjected to overnight exposure to primary antibody against collagen I or IV (Abcam Ltd, MA, USA). Membranes were also probed with the loading control, α-tubulin (Sigma, MO, USA). The membranes were then washed and exposed to anti-rabbit horseradish peroxidase-conjugated secondary antibody for 1 hr. After 30 min of washing, antibody binding was detected using the ECL Advance Western Blotting detection kit and quantitated by densitometry using the program Quantity One (Bio-Rad, Hercules, CA, USA) and standardised for loading with α-tubulin. Data are expressed as fold change to control (CTL). Three independent experiments were analysed (n = 3).

### Statistical Analyses

The data are expressed as mean ± standard error of mean (SEM). Comparison between groups were analysed by performing one-way ANOVA with Newman-Keuls post hoc test. All statistical analyses were performed using GraphPad Prism version 5.0 (GraphPad Software, La Jolla, CA, USA). A *P* value <0.05 was considered statistically significant.

## Results

### In vitro Assessments

#### HAEC

**Figure 2:**
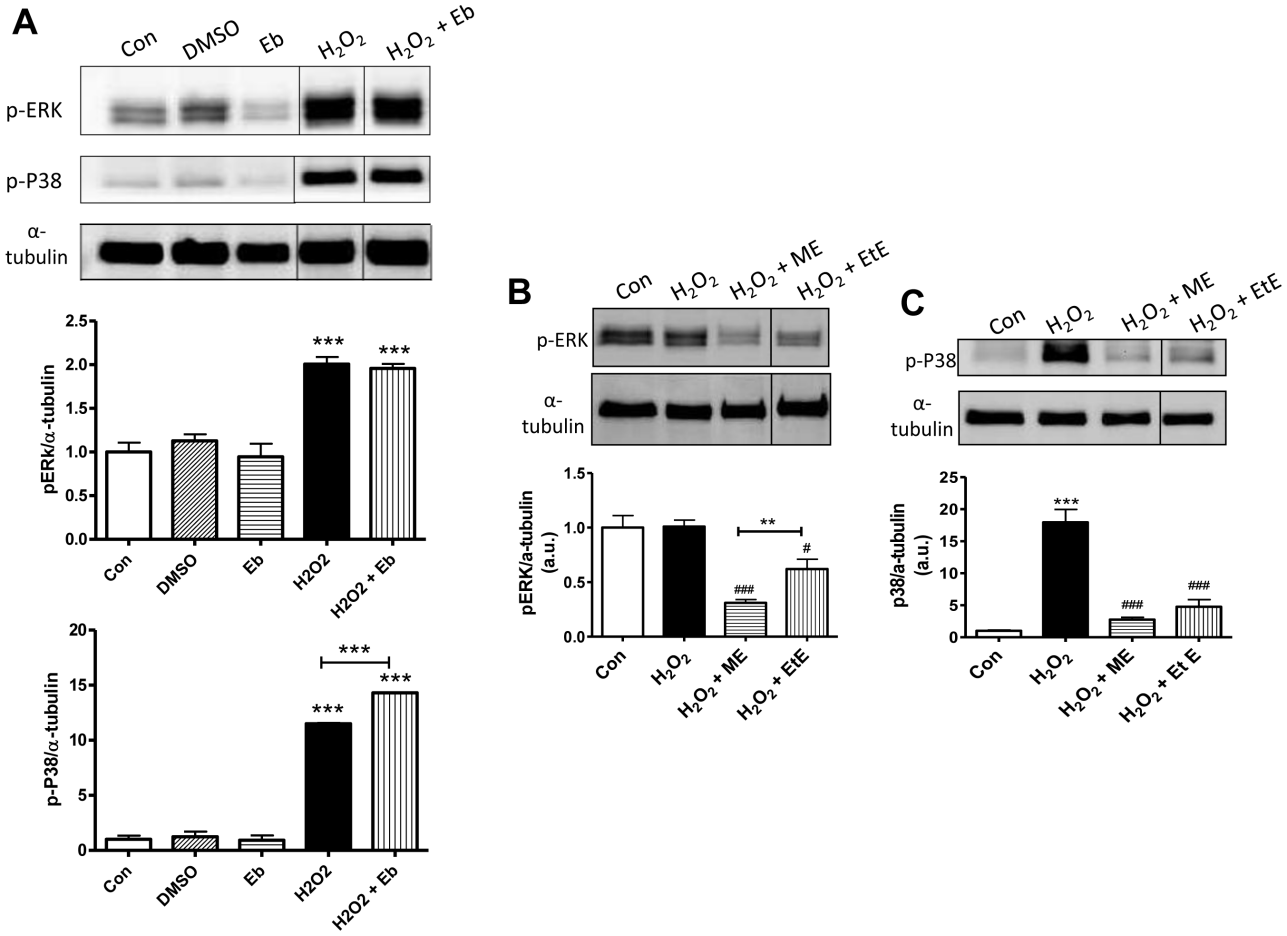
ERK and P38 phosphorylation in HAECs treated with hydrogen peroxide and ebselen or its analogues. **A)** 200 µM hydrogen peroxide (H_2_O_2_) treatment of human aortic endothelial cells (HAEC) results in a ∼2-fold increase in ERK phosphorylation and a ∼10-fold increase in P38 phosphorylation. Treatment with ebselen at 0.01 µM does not affect the H_2_O_2_–induced phosphorylation of either ERK or P38. ***P<0.001 vs control (con), DMSO and ebselen (Eb) treated cells. **B)** Treatment of HAEC with m-hydroxy ebselen (ME) or ethanol ebselen (EtE) results in a ∼75% and 50% reduction in ERK phosphorylation (pERK), respectively. In this experiment, basal levels for pERK were elevated in control cells most likely as a result of the later passage of these cells. ^###^ *P*<0.001 and ^#^ *P*<0.05 vs H_2_O_2_ treated cells. **P<0.01 EtE vs ME. **C)** 200 µM H_2_O_2_ treatment of HAEC results in a ∼15-fold increase in P38 phosphorylation. Treatment with ME or EtE at 0.01 µM significantly reduced the H_2_O_2_-induced phosphorylation of P38. ***P<0.001 vs control (con), ^###^ *P*<0.001 vs H_2_O_2_ treated cells.

Treatment of HAEC with 200 µM H_2_O_2_ resulted in significant increases (*P*<0.001) in the phosphorylation of ERK and P38 compared with controls. Treatment with Eb did not alter the amount of hydrogen peroxide-mediated phosphorylation of ERK or P38 (Figure 2A). ME and EtE significantly reduced the level of hydrogen peroxide-induced phosphorylation of P38 (*P*<0.001) as well as lowering the phosphorylation of ERK (*P*<0.001 for ME and *P*<0.05 for EtE vs H_2_O_2_-treated cells; Figure 2B and C). Importantly, the reduction in ERK phosphorylation was significantly greater (*P*<0.01) in the presence of ME (∼71%) than that observed after treatment with EtE (∼40%). Similarly, ME caused a greater inhibition of P38 phosphorylation (∼85%) than that caused by EtE (∼73%) although there was no significant difference between the two treatment groups. These data suggested in *in vitro* experiments at least that Eb had no inhibitory effect on these two important pro-inflammatory mediators, whilst ME showed greater inhibitory potential than EtE.

### Clinical and Metabolic Parameters

At the end of the treatment period, all diabetic groups displayed significant reductions in body weight consistent with our previous results [24], [25] and the known effects of diabetes in these mice. Throughout the study, diabetic dKO mice displayed significantly elevated blood glucose levels (data not shown) and on termination had significantly increased blood glucose and glycated haemoglobin levels when compared with non-diabetic dKO mice (*P*<0.001, Table 1). Glucose and glycated haemoglobin were not affected by Eb or ME treatment (Table 1). Consistent with our previous study in diabetic dKO mice [25], there were no significant differences in total cholesterol, HDL and LDL levels in all experimental groups. All diabetic groups displayed increased kidney weight to body weight ratios most likely as a consequence of the diabetes-induced reductions in body weight (Table 1).

**Table 1:** Basic characteristics (clinical parameters).

	ND (n = 9)	ND+ME (n = 6)	Diab (n = 5)	Diab+Eb (n = 8)	Diab+ME (n = 6)
**BW,** g	28.8±0.5	27.7±0.7	24.2±0.9**	23.1±1.1***	23.0±0.8**
**HbA1c,** %	5.2±0.2	4.7±0.2	17.0±1.0***	NA	18.0±0.6***
**Blood glucose,** mmol/L	12.2±1.0	8.3±1.0	28.6±2.9***	24.2±2.0***	26.6±1.9***
**Total cholesterol,** mmol/L	5.8±0.5	5.5±0.4	10.4±2.2	8.2±1.2	7.1±0.7
**Triglycerides,** mmol/L	1.9±0.5	2.0±0.2	3.5±0.8	3.4±0.5	3.2±0.4
**HDL,** mmol/L	1.2±0.1	1.2±0.1	2.2±0.5	1.7±0.3	1.3±0.2
**LDL,** mmol/L	3.7±0.3	3.4±0.3	6.6±1.5	5.0±0.8	4.3±0.5
**R.Kid weight:BW,** mg/g	7.0±0.2	6.5±0.1	10.2±0.5***	10.0±0.5***	10.5±0.2***
**L.Kid weight:BW,** mg/g	7.1±0.3	6.6±0.2	10.1±0.6***	9.8±0.4***	10.2±0.3***
**UACR,** mg/mmol	20.9±3.3	18.1±2.8	65.0±11.6**	51.2±9.3*	39.9±12.1

* *P*<0.05;

** *P*<0.01;

*** *P*<0.001 vs non-diabetic ApoE/GPx1 dKO mice.

ND, non-diabetic; Diab, diabetic; ME, m-hydroxy ebselen; Eb, ebselen; BW, body weight; HbA1c, glycated haemoglobin; HDL, high-density lipoprotein; LDL, low-density ± SEM. lipoprotein; UACR, urinary albumin to creatinine ratio; NA, not assessed. Data are presented as mean.

Diabetes caused an increase in urinary albumin to creatinine (UACR) ratio (*P*<0.01, Table 1) in dKO mice. Twelve weeks of ME gavage resulted in a greater reduction in UACR in the diabetic dKO mice than Eb, although this did not reach significance when compared to vehicle-treated diabetic mice (Table 1).

### Atherosclerotic Lesions are Attenuated by ME

#### En face

**Figure 3:**
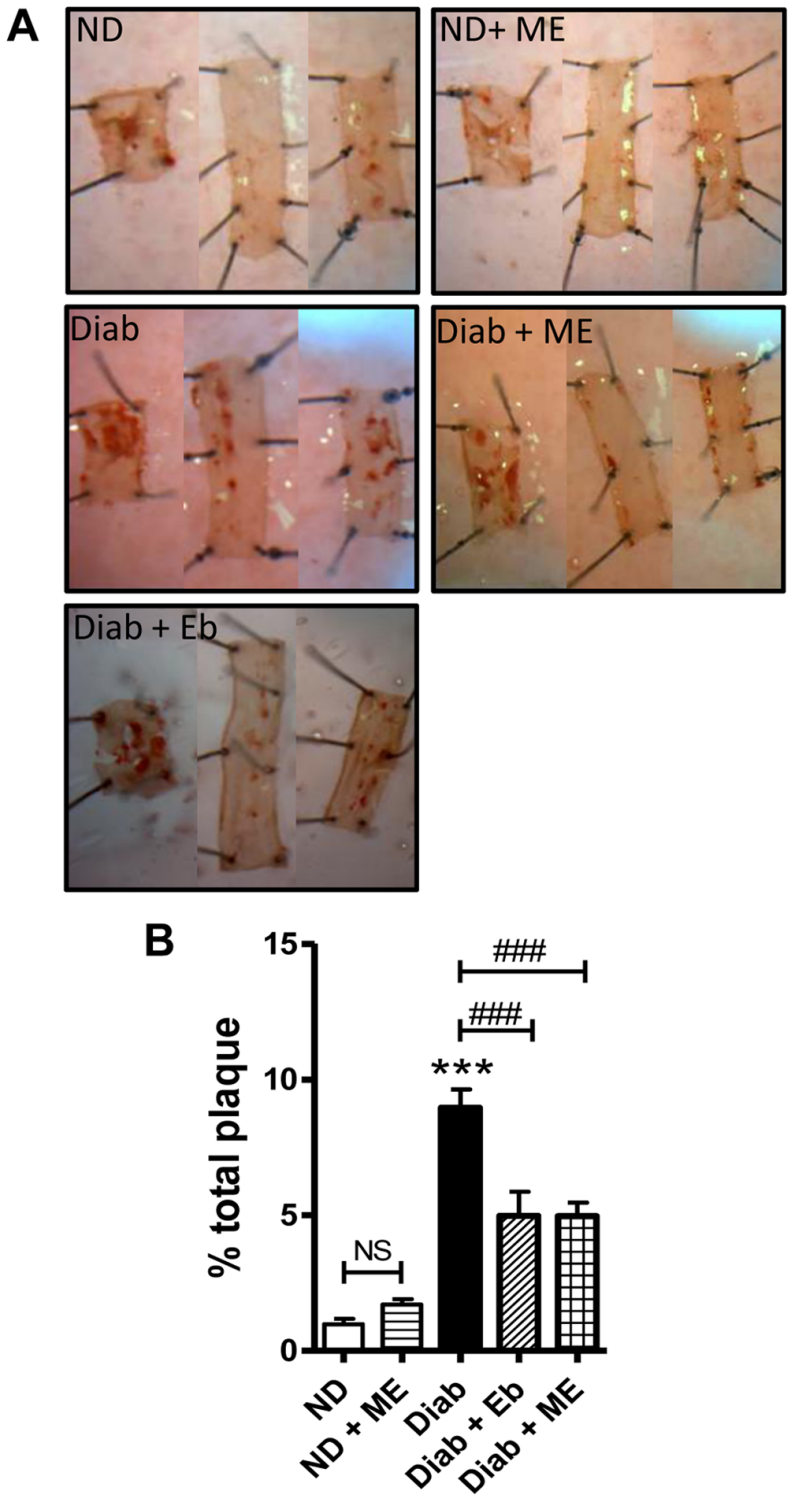
Diabetes-associated atherosclerosis is attenuated in the aorta by the ebselen analogue, m-hydroxy ebselen. 14 weeks of diabetes resulted in a significant ∼8-fold increase in plaque (stained red) within the aorta of ApoE/GPx1 dKO mice. Treatment with both m-hydroxy ebselen (ME) and ebselen (Eb) at 10 mg/kg twice daily significantly attenuated atherosclerotic plaque deposition within the diabetic aorta. ***P<0.001 vs ND control; ^###^ *P*<0.001 vs diabetic aorta; NS = not significant. ND = non-diabetic; ME = m-hydroxy ebselen; Diab = diabetic; Eb = ebselen.

Diabetes induced an approximately 9-fold increase in total plaque formation in the dKO aortas, which was significantly (*P*<0.001∶45% reduction in plaques) attenuated by ME. Eb also significantly attenuated total aortic plaque deposition by approximately 45% (Figure 3).

#### Aortic sinus lesions

**Figure 4:**
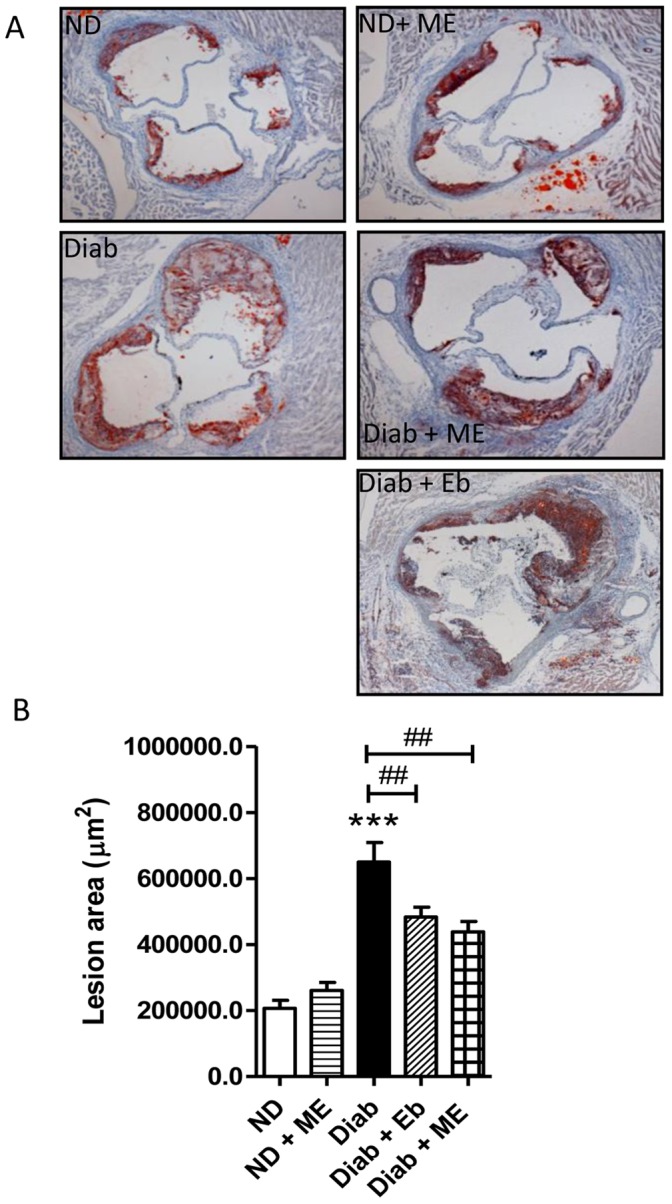
Diabetes-associated atherosclerosis is attenuated in the aortic sinus region by the ebselen-analogue, m-hydroxy ebselen. Aortic sinus regions of ApoE/GPx1 dKO mice were stained with Oil Red O to detect plaque after 14 weeks of diabetes. Diabetes resulted in a significant ∼4-fold increase in plaque within the aorta sinus region. Treatment with both m-hydroxy ebselen (ME) and ebselen (Eb) at 10 mg/kg twice daily significantly attenuated atherosclerotic plaque within the sinus. Although we had previously observed no reduction in aortic sinus lesions after 10 and 20 weeks of ebselen treatment at equivalent concentrations in diabetic ApoE/GPx1 dKO mice [25], we believe that the current study most likely reflects a reduction in the non-lipid components of the plaque by Eb since the entire plaque area was taken into account in the measurement of plaque area in the current study, whilst Oil Red O positive staining for lipid was measured previously. ***P<0.001 vs ND control; ^##^ *P*<0.01 vs diabetic sinus. ND = non diabetic; ME = m-hydroxy ebselen; Diab = diabetic; Eb = ebselen.

Diabetes significantly increased lesion deposition within the aortic sinus of dKO mice by approximately 3-fold after 14 weeks of diabetes (*P*<0.001 vs ND mice aortic sinuses; Figure 4). This increase was significantly attenuated (∼33%; *P*<0.01 vs Diab group) by treatment with ME. Eb also significantly attenuated lesions by ∼26% (*P*<0.01) compared with that seen in the diabetic group, albeit to a lesser extent than ME, although this difference between treatment groups was not significantly different.

### Aortic Oxidative Stress is Attenuated by ME

**Figure 5:**
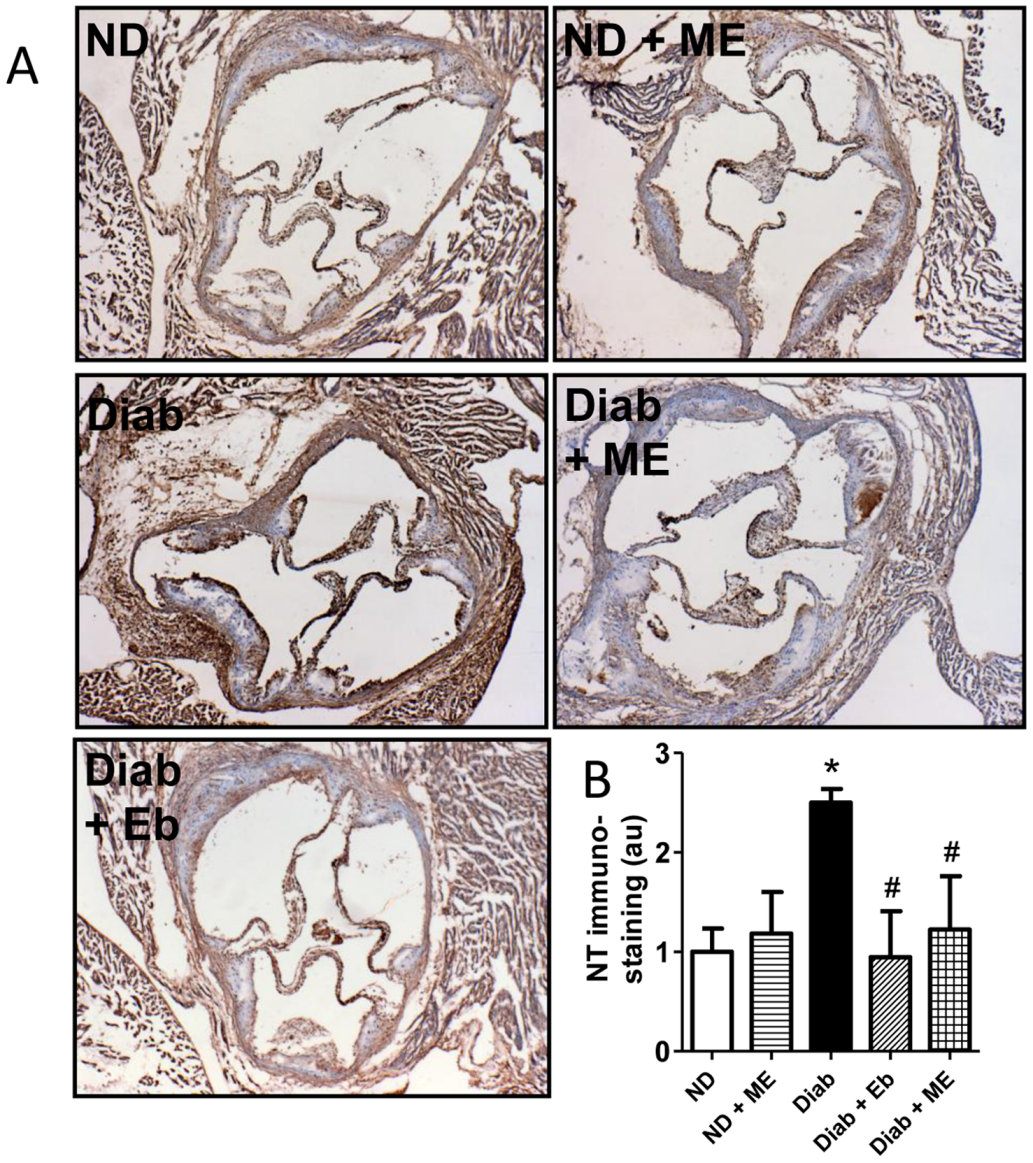
Oxidative stress is reduced in the aortic sinus region after treatment with m-hydroxy ebselen. Aortic sinus regions of ApoE/GPx1 dKO mice were immunostained with antibody to nitrotyrosine to detect oxidative-modified proteins. Diabetes resulted in a significant ∼2-fold increase in nitrotyrosine (NT) staining within the aorta sinus region of dKO mice. Treatment with m-hydroxy ebselen (ME) and ebselen (Eb) significantly attenuated the level of NT staining observed within the aortic sinus region. *P<0.05 vs ND control; ^#^ *P*<0.05 vs diabetic sinus. ND = non diabetic; ME = m-hydroxy ebselen; Diab = diabetic; Eb = ebselen.

Diabetes caused a significant increase in the amount of nitrotyrosine immunostaining in the aortic sinus region of ApoE/GPx1 dKO mice (*P*<0.05). This was significantly attenuated by both ME and Eb treatment although there was no significant difference between the two treatment groups (Figure 5).

### Kidney Morphology

#### Tubulointerstitial injury

**Figure 6:**
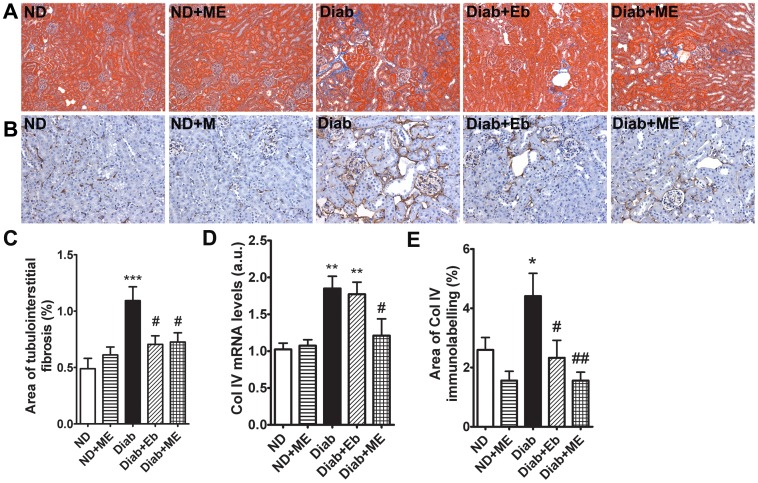
Tubulointerstitial fibrosis is reduced in the kidneys of diabetic ApoE/GPx1 dKO mice after treatment with m-hydroxy ebselen. **A)** ApoE/GPx1 dKO kidneys were stained with Masson’s trichrome (stained blue) to detect tubulointerstitial matrix proteins. Diabetic dKO kidneys were associated with a significant increase in tubulointerstitial fibrosis and this increase was attenuated by both ebselen (Eb) and m-hydroxy ebselen (ME). **B)** Collagen IV immunostaining (stained brown) was increased in diabetic dKO kidneys and attenuated by both ME and Eb. **C)** Quantitation of tubulointerstitial fibrosis within the kidney cortex after Masson’s trichrome staining. **D)** Quantitative RT-PCR analysis revealed a significant increase in collagen IV mRNA expression in the diabetic dKO kidney cortex which was significantly attenuated by ME. **E)** Quantitation of collagen IV immunostaining within the kidney cortex. *P<0.05, **P<0.01, ***P<0.001 vs ND control; ^#^ *P*<0.05, ^##^ *P*<0.01 vs diabetic kidney. ND = non diabetic; ME = m-hydroxy ebselen; Diab = diabetic; Eb = ebselen; Col IV = collagen IV.

Masson’s trichrome staining demonstrated an increased tubulointerstitial fibrosis in the diabetic dKO mice kidney when compared to non-diabetic controls (*P*<0.001, Figure 6). Treatment of Eb and ME in diabetic animals was associated with significant reduction in the accumulation of matrix in the tubulointerstitium (*P*<0.05, Figure 6). Collagen IV has been shown to contribute to diabetic tubulointerstitial matrix accumulation [28]. In an attempt to further quantitate the changes observed after Masson’s trichrome staining, we evaluated the effects of Eb and ME on the protein and gene expression of this known fibrotic mediator. As expected, we observed that the mRNA and protein expression of collagen IV was significantly increased in the kidney cortex of diabetic dKO mice (*P*<0.01 and *P*<0.05, respectively, Figure 6). Importantly, ME significantly attenuated both collagen IV mRNA and protein expression. Interestingly, in this instance, Eb significantly reduced the protein expression of collagen IV but failed to reduce its mRNA expression, suggesting that the mechanism of action by which Eb reduced collagen IV in the kidney may be post-transcriptional.

#### Glomerular injury

**Figure 7:**
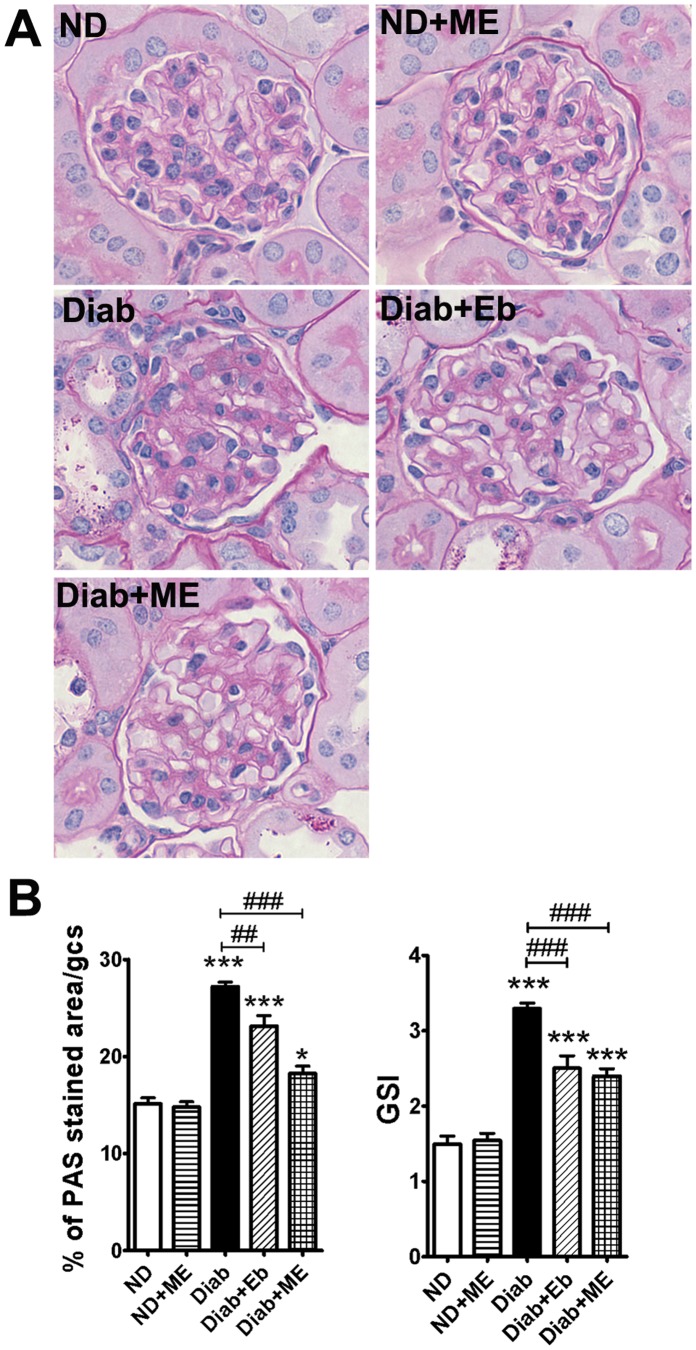
Glomerulosclerosis is attenuated in the kidneys of diabetic ApoE/GPx1 dKO mice after treatment with m-hydroxy ebselen. Glomerulosclerosis was assessed by measuring Periodic acid-Schiff (PAS)-stained area (stained dark magenta) per glomerulus (indicative of mesangial expansion) and also scored for glomerulosclerosis index (GSI). Both mesangial expansion and GSI were significantly increased in the diabetic kidneys. Treatment with m-hydroxyebselen (ME) and ebselen (Eb) significantly reduced mesangial expansion and GSI in diabetic dKO kidneys. *P<0.05, ***P<0.001 vs ND control; ^##^ *P*<0.01, ^###^ *P*<0.001 vs diabetic kidney. ND = non diabetic; ME = m-hydroxy ebselen; Diab = diabetic; Eb = ebselen.

The percentage of PAS-positive material, indicative of mesangial expansion, was significantly increased in the diabetic dKO mice (*P*<0.001, Figure 7). Furthermore, the glomerulosclerosis index (GSI) of the diabetic dKO mice was significantly higher than non-diabetic controls (*P*<0.001, Figure 7). Both the mesangial expansion and glomerulosclerosis were attenuated in Eb and ME-treated diabetic dKO mice (Figure 7). Interestingly, the reduction in PAS staining was more significant after treatment with ME (∼35%; *P*<0.001) than with Eb (∼15%; *P*<0.01) compared to non-treated diabetic glomeruli.

**Figure 8:**
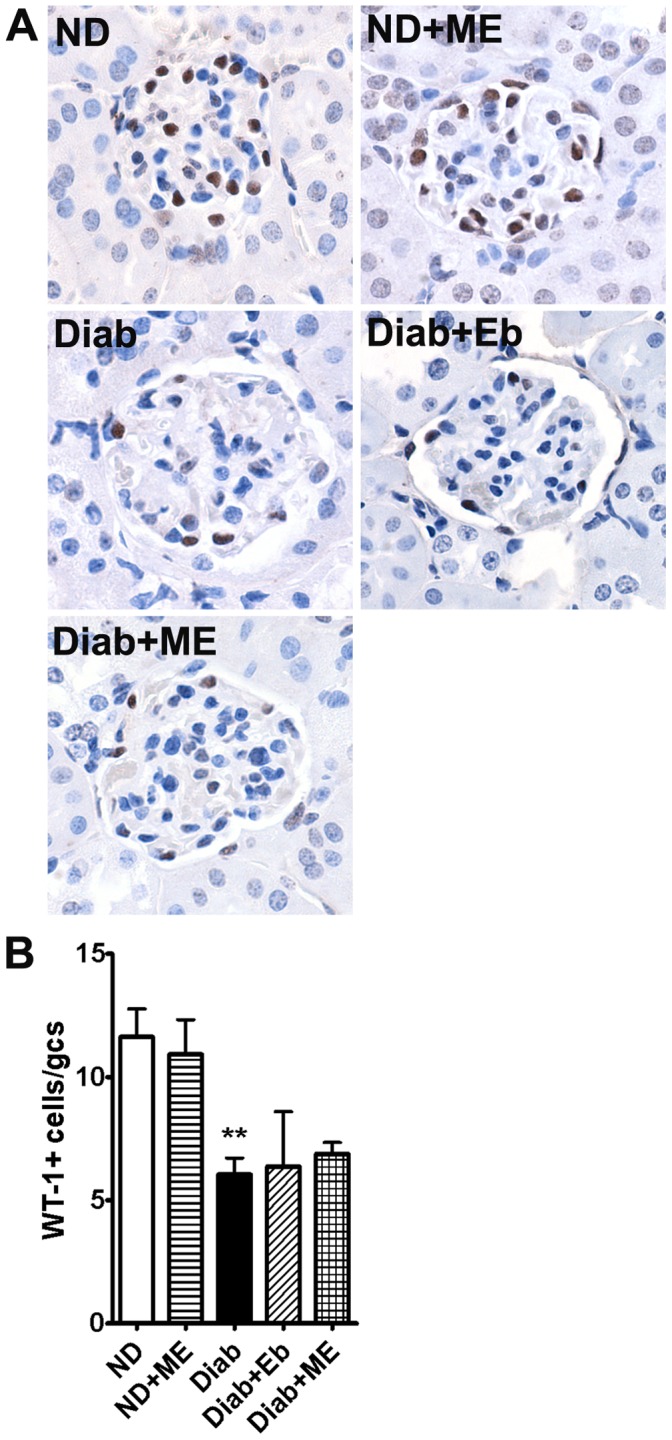
Podocyte loss is not attenuated in the kidneys of diabetic ApoE/GPx1 dKO mice after treatment with m-hydroxy ebselen. Podocyte number as counted by WIlms Tumor-1 (WT-1) positive nuclei (brown nuclei staining) per glomerulus was significantly reduced in the diabetic kidneys. Both ebselen (Eb) and m-hydroxy ebselen (ME) treatment did not prevent the loss of podocyte in diabetic dKO mice. **P<0.01 vs ND control. ND = non diabetic; ME = m-hydroxy ebselen; Diab = diabetic; Eb = ebselen; WT-1 = Wilms Tumour-1.

Podocyte injury, which manifests as a decrease in the number of podocytes per glomerulus, is reported to be the strongest predictor of the progression of albuminuria [29]. Since glucose-induced ROS have been shown to be a major contributor to podocyte apoptosis and depletion in experimental diabetes leading to diabetic nephropathy [30], we also measured the extent of WT-1 staining (a podocyte-specific nuclei marker) in the glomeruli of all groups in this study. As shown in Figure 8, diabetes caused a significant reduction in podocyte number in the glomeruli of dKO mice and may have contributed to the significant increase in the UACR of the diabetic group as shown in Table 1. However, after 12 weeks of treatment, Eb and ME did not protect the diabetic dKO mice from podocyte depletion since the number of WT-1 positive cells was not significantly different from the diabetic group. This result is in agreement with a previous study showing that selenoproteins may not be important in the protection of podocyte-specific injury in diabetic nephropathy [31].

### Oxidative Stress within the Kidney is Attenuated by ME

**Figure 9:**
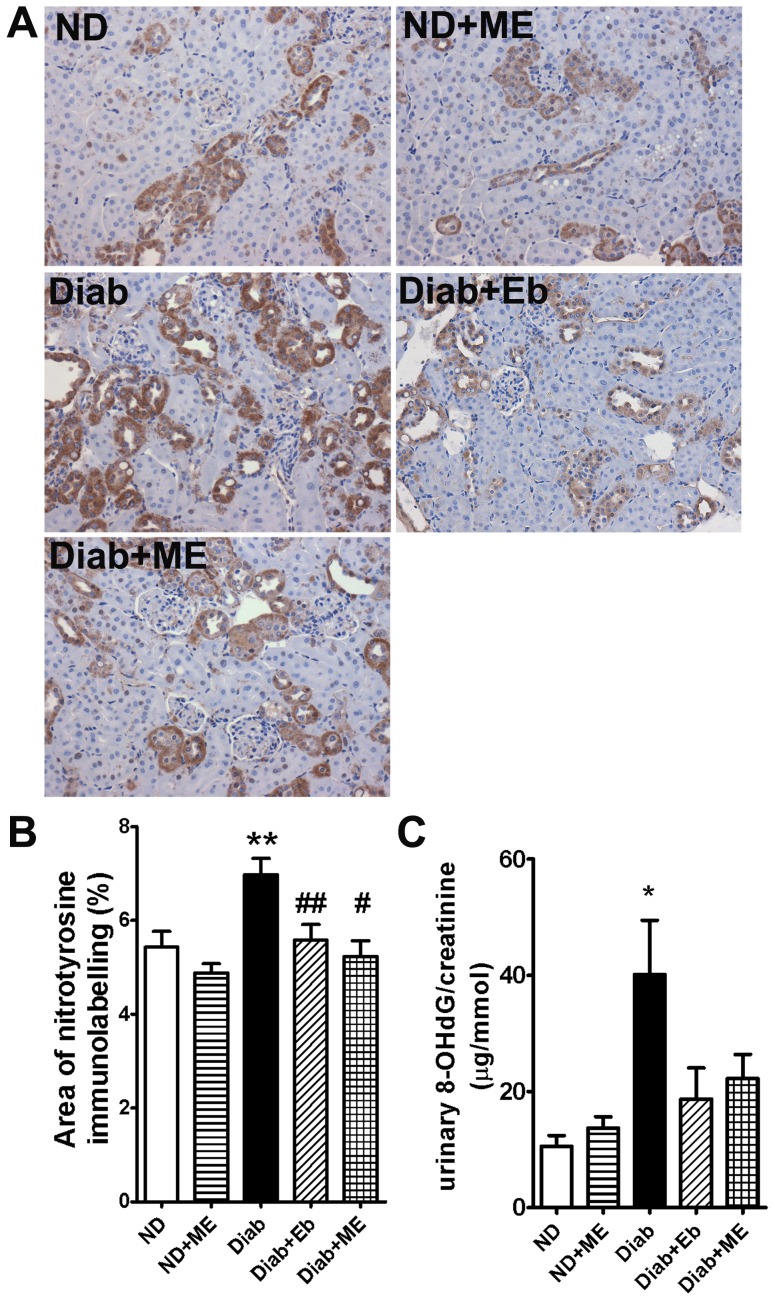
Oxidative stress is reduced in the kidneys of diabetic ApoE/GPx1 dKO mice after treatment with m-hydroxy ebselen. **A)** Kidneys of dKO mice were immunostained with antibody to nitrotyrosine (stained brown) to detect peroxynitrite-induced damage to the tissue. Diabetes resulted in an increase in nitrotyrosine immunostaining, particularly within the tubules. **B)** Histogram showing that both m-hydroxy ebselen (ME) and ebselen (Eb) treatment attenuated nitrotyrosine immunolabelling in the tubules of diabetic dKO kidneys. **C)** Histogram showing the urinary levels of 8-hydroxy-2-deoxy guanosine (8-OHdG) measured by an EIA kit. Values are normalised to urinary creatinine levels and expressed as µg/mmol. *P<0.05, **P<0.01 vs ND control; ^#^ *P*<0.05, ^##^ *P*<0.01 vs diabetic kidney. ND = non diabetic; ME = m-hydroxy ebselen; Diab = diabetic; Eb = ebselen.

Oxidative stress-induced damage in the kidney was assessed by measuring the levels of nitrotyrosine immunolabelling. Diabetic kidneys displayed significantly increased levels of nitrotyrosine when compared to non-diabetic controls (*P*<0.01, Figure 9). Treatment with both Eb and ME significantly reduced the levels of nitrotyrosine staining in the diabetic dKO kidneys (Figure 9B). Furthermore, urinary 8-OHdG, which is a marker for oxidative DNA damage, was significantly increased in the diabetic dKO mice (Figure 9C). Treatment with ME and Eb reduced the levels of urinary 8-OHdG by approximately 50% when compared to the non-treated diabetic mice, albeit that these changes did not reach significance. Importantly, the levels of 8-OHdG detected in both ME and Eb treated diabetic groups were not significantly different from non-diabetic levels, suggesting that both treatments reduced oxidative DNA damage in diabetic mice.

### The Bioactivity of TGF-β in the Diabetic Kidney is Attenuated by ME

**Figure 10:**
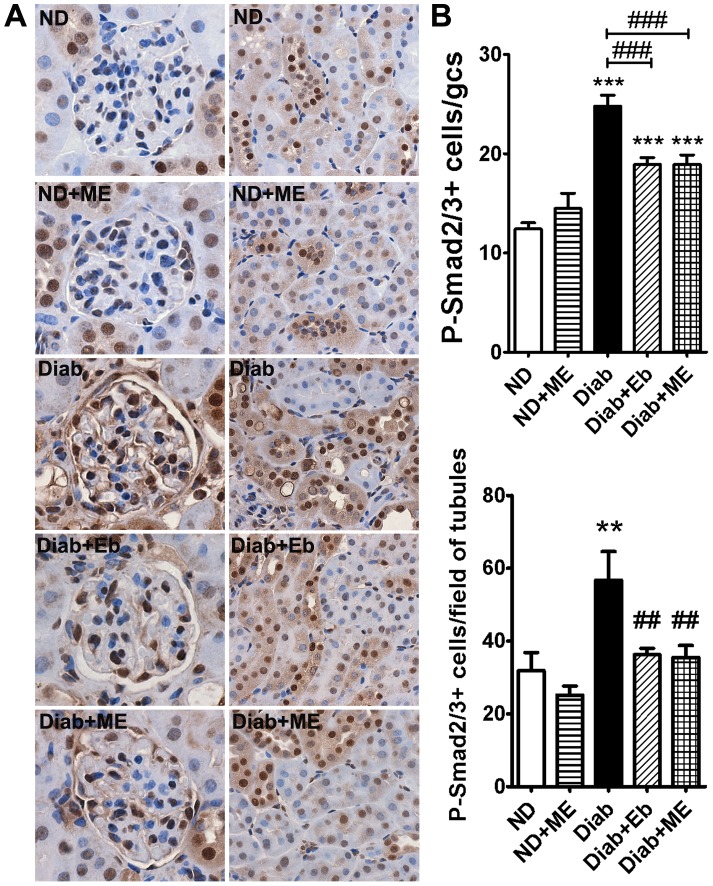
Bioactivity of the profibrotic cytokine, transforming growth factor-β (TGF-β) is reduced in the kidneys of diabetic ApoE/GPx1 dKO mice after treatment with m-hydroxy ebselen. The bioactivity of TGF-β was assessed by measuring the activation (phosphorylation) of its downstream mediator, Smad2. Diabetes induced a significant increase in the bioactivity of TGF-β in both the glomeruli and tubules (positive brown nuclei staining) of the kidney. Treatment with m-hydroxy ebselen (ME) and ebselen (Eb) attenuated the increase in TGF-β activation to a similar extend in the diabetic dKO kidney. **P<0.01, ***P<0.001 vs ND control; ^##^ *P*<0.01, ^###^ *P*<0.001 vs diabetic kidney. ND = non diabetic; ME = m-hydroxy ebselen; Diab = diabetic; Eb = ebselen.

The activation of TGF-β was assessed by measuring its downstream mediator, phosphorylated Smad2/3. There was a significant increase in the number of cells stained positive for phosphorylated Smad2/3 in the diabetic dKO kidney, in both the glomeruli and tubules (*P*<0.001 and *P*<0.01, respectively, Figure 10). Both Eb and ME attenuated the activation of TGF-β signalling in the glomeruli and tubules to a similar extend in the diabetic mice.

### Collagen I and IV Expression is Attenuated by ME in NRK Cells

**Figure 11:**
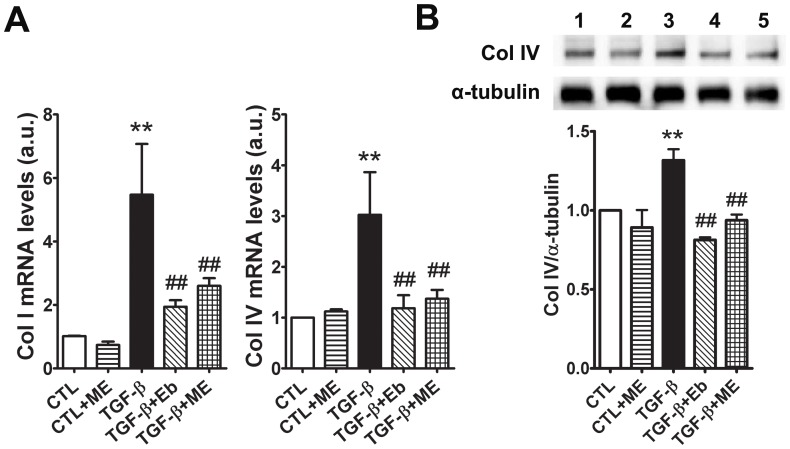
Collagen I and IV expression in rat kidney tubular cells (NRK52E) after stimulation with transforming growth factor-β_1_ and treatment with m-hydroxy ebselen. **A) After 72 hours of stimulation with TGF-β_1_, the gene expression of collagen I and collagen IV was measured by qRT-PCR and found to be significantly increased in NRK52E cells.** Treatment with ME significantly reduced the TGF-β_1_-stimulated increase in collagen I and IV gene expression in NRK52E cells, with similar effects observed in cells treated with Eb. B) Western blot analysis demonstrated increased expression of collagen IV in TGF-β_1_-treated NRK52E cells and this was significantly attenuated by Eb and ME. Data were collected from three independent experiments (n = 3) and standardised for loading with α-tubulin and expressed as a fold change to control (CTL). Lane 1 = CTL+DMSO; lane 2 = CTL+ME; lane 3 = TGF-β_1_+DMSO; lane 4 = TGF-β_1_+Eb; lane 5 = TGF-β_1_+ME. **P<0.01 vs CTL; ^##^ *P*<0.01 vs TGF-β_1_-stimulated cells. CTL = control; TGF-β = transforming growth factor-β; ME = m-hydroxy ebselen; Eb = ebselen.

When NRK52E cells were stimulated with TGF-β_1_ for 72 hours, collagen I and IV mRNA levels were significantly increased in agreement with previous data (Figure 11) [32]. Treatment of ME at 2.5 µM significantly attenuated TGF-β_1_-stimulated increase in collagen I and IV gene expression (*P*<0.01, Figure 11A) compared to DMSO-treated cells. Treatment of NRK52E cells with Eb reduced collagen I and IV gene expression to the same extent as ME. Furthermore, Western blot analysis demonstrated that collagen IV protein expression was increased in TGF-β_1_-treated NRK52E cells, and that both Eb and ME significantly reduced the TGF-β_1_-induced collagen IV protein expression in these cells (*P*<0.01, Figure 11B). In addition, both ME and Eb reduced collagen I protein levels by approximately 35% after stimulation with TGF-β (P<0.01 and P = 0.05 for ME and Eb, respectively; data not shown).

### Discussion and Conclusions

It has been postulated by us [23], [33] and others [34], [35] that synthetic modifications to enhance the nucleophilic attack by thiols on the sulphur atom of the selenenyl sulphide form of Eb, would improve its efficacy to act as a radical scavenging compound. We had previously extensively characterised a diverse range of GPx mimetics using computational, physicochemical and biochemical methods [23], [33]. These studies demonstrated that two compounds, ME and EtE, had properties which were consistent with being significantly more effective GPx mimetics than Eb [23]. As reported in this study, it became important to then examine the biological activity of these compounds using cell based and *in vivo* models of GPx activity, to determine if such differences translate into effective improvements in GPx performance. Indeed, to date, there have been only limited *in vitro* and *in vivo* comparison of GPx mimetic compounds [36]. The results of this study have shown several important aspects relating to the testing of modified Eb analogues in cell based and *in vivo* models.

Firstly, our study has shown that placement of an electron withdrawing 3-hydroxy moiety on the phenyl ring of Eb renders the derivative compound, ME, more efficacious than Eb in lowering two pro-inflammatory signalling mediators in cell based assays. Indeed, where Eb had no effect on the phosphorylation status of the two mediators, P38 and ERK, the derivative compound ME significantly reduced the phosphorylation of P38 and ERK in human aortic endothelial cells. These results suggest that this derivative may be more effective than Eb in lessening the activation of these two pro-inflammatory mediators known to play significant roles in promoting atherogenesis, possibly via its antioxidant function in removing RO/NS [9], [11].

Our results also showed that replacing the N-phenyl group of Eb with a N-(2-hydroxyethyl) substituent renders the derivative compound, EtE, more efficacious than Eb in lowering pro-inflammatory signalling in our cell based assays. However, ME was found to be more efficient than EtE, particularly with respect to the attenuation of ERK phosphorylation. Based on our previous *in vitro* studies showing the superior activity of ME compared with either EtE or Eb to (1) inhibit the peroxynitrite-mediated nitration of L-tyrosine in biochemical assays [20]; (2) reduce hydrogen peroxide and organic peroxide in the presence of glutathione [23] and (3) together with the new data from this study showing its greater effectiveness in limiting two pro-inflammatory mediators *in vitro*, we selected ME for further testing in our *in vivo* diabetic ApoE/GPx1 dKO mouse model. We have previously shown that 20 weeks of diabetes significantly enhances pro-inflammatory and pro-atherogenic mediators as well as atherosclerosis [24] and diabetic kidney disease [25] in ApoE/GPx1 dKO mice. Furthermore, our recent study showed that Eb significantly attenuates pro-inflammatory and pro-atherosclerotic mediators as well as diabetes-associated atherosclerosis and diabetic nephropathy after 20 weeks of treatment in the diabetic ApoE/GPx1 dKO mice [25].

In this study, we now confirm and expand on our initial findings by demonstrating that ME, in an analogous fashion to the parent compound Eb, reduces oxidative stress and diabetes-associated atherosclerosis in the diabetic ApoE/GPx1 dKO mouse. Furthermore, ME also lowered diabetes-associated oxidative stress within the interstitial region of the diabetic dKO kidney. Previous studies have shown that ROS are implicated in TGF-β-mediated tubulointerstitial fibrosis and glomerulosclerosis in diabetic nephropathy [37]. Consistent with previous studies [38], we now show a correlative reduction in oxidative stress, and a reduction in the activity of the profibrotic cytokine, TGF-β, as well as reductions in tubulointerstitial fibrosis and glomerulosclerosis within the diabetic kidney through the use of Eb and its analogue. In addition, ME was also effective in reducing albuminuria, the functional readout of diabetic kidney disease. Moreover, the notion that ROS play an important role in TGF-β signalling was further strengthened in our *in vitro* experiments using NRK52E cells where the antioxidants, Eb and ME, significantly reduced TGF-β-stimulated collagen expression.

However, this study has demonstrated that there was mostly no additional benefit derived from the modified Eb compound with respect to further reductions in either structural or functional readouts for DN or diabetes-associated-atherosclerosis over that noted by the parent compound Eb, despite significant improvements for ME versus that of Eb in biochemical and *in vitro* (cell) based assays. We did however observe a slight improvement in the extent of albuminuria and a greater reduction in mesangial expansion after ME treatment when compared to Eb, suggesting that for this parameter at least, ME has shown better protection against diabetic mesangial injury than Eb. Interestingly, our study has additionally shown that neither Eb nor ME protected against podocyte loss in diabetic mice, thereby eliminating the podocyte as a potential protective mechanism of ME. Our study therefore highlights the importance of *in vivo* studies in selecting more efficacious Eb analogues since our data demonstrate that biochemical and *in vitro* assays may only partly anticipate the *in vivo* situation.

In conclusion, this study has demonstrated the effectiveness of a modified Eb analogue, ME, in lowering end-points of two often linked diabetic complications. These results are in agreement with our previous study using the parent compound Eb and therefore strengthen the notion that bolstering GPx-like activity may be a useful therapeutic strategy to lessen the burden of complications in diabetic patients. This is particularly relevant since clinical findings have shown that reductions in GPx1 activity have been linked to increased cardiovascular disease, both within a diabetic setting and in association with coronary artery disease [39], [40], [41]. The development of efficacious GPx1 mimetics is therefore of significant clinical relevance in the quest for developing targeted antioxidant treatments to treat diabetic complications. This study has also highlighted the importance of *in vivo* testing of modified Eb analogues, since *in vitro* and cell based assays cannot be relied upon to predict the *in vivo* effectiveness of the Eb analogues.
